# Neisseria cinerea Expresses a Functional Factor H Binding Protein Which Is Recognized by Immune Responses Elicited by Meningococcal Vaccines

**DOI:** 10.1128/IAI.00305-17

**Published:** 2017-09-20

**Authors:** Hayley Lavender, Katy Poncin, Christoph M. Tang

**Affiliations:** Sir William Dunn School of Pathology, University of Oxford, Oxford, United Kingdom; University of California, Davis

**Keywords:** Neisseria cinerea, Neisseria meningitidis, complement, fHbp, vaccine

## Abstract

Neisseria meningitidis is a major cause of bacterial meningitis and sepsis worldwide. Capsular polysaccharide vaccines are available against meningococcal serogroups A, C, W, and Y. More recently two protein-based vaccines, Bexsero and Trumenba, against meningococcal serogroup B strains have been licensed; both vaccines contain meningococcal factor H binding protein (fHbp). fHbp is a surface-exposed lipoprotein that binds the negative complement regulator complement factor H (CFH), thereby inhibiting the alternative pathway of complement activation. Recent analysis of available genomes has indicated that some commensal Neisseria species also contain genes that potentially encode fHbp, although the functions of these genes and how immunization with fHbp-containing vaccines could affect the commensal flora have yet to be established. Here, we show that the commensal species Neisseria cinerea expresses functional fHbp on its surface and that it is responsible for recruitment of CFH by the bacterium. N. cinerea fHbp binds CFH with affinity similar to that of meningococcal fHbp and promotes survival of N. cinerea in human serum. We examined the potential impact of fHbp-containing vaccines on N. cinerea. We found that immunization with Bexsero elicits serum bactericidal activity against N. cinerea, which is primarily directed against fHbp. The shared function of fHbp in N. cinerea and N. meningitidis and cross-reactive responses elicited by Bexsero suggest that the introduction of fHbp-containing vaccines has the potential to affect carriage of N. cinerea and other commensal species.

## INTRODUCTION

The genus Neisseria includes two major human pathogens, Neisseria meningitidis and Neisseria gonorrhoeae (1, 2). N. meningitidis is a commensal of the human nasopharynx in approximately 10 to 40% of the general population but can traverse the epithelial layer and cause systemic disease (3), while the gonococcus is a leading cause of sexually transmitted disease (4). In contrast, nonpathogenic Neisseria species are ubiquitous commensals of the human oral cavity and nasopharynx in healthy individuals and constitute approximately 10% of the human oral bacterial flora (1, 5, 6). Even though Neisseria polysaccharea, Neisseria lactamica, and Neisseria cinerea are closely related to the meningococcus and the gonococcus (7, 8), there are only rare case reports of invasive disease caused by these commensal species, and they are usually limited to immunocompromised hosts (9).

N. meningitidis can be categorized into 13 serogroups based on the structure of its polysaccharide capsule, with 6 serogroups (A, B, C, W, X, and Y) responsible for the majority of disease worldwide (3, 10–13). Capsular polysaccharide conjugate vaccines have been developed to protect individuals against disease caused by some serogroups of N. meningitidis (i.e., A, C, W, and Y), while outer membrane vesicle (OMV) vaccines have been successfully employed to combat epidemic disease caused by single strains (14, 15). However, these approaches cannot prevent endemic serogroup B N. meningitidis disease, which is still a significant cause of morbidity and mortality in Europe and the United States (3). This is because immunization with the serogroup B capsule, which is identical to a modification on NCAM-1 (16), can induce autoimmunity, while OMV vaccines offer only limited cross protection against diverse strains (17–20).

Two recently licensed serogroup B vaccines, Bexsero and Trumemba, contain factor H binding protein (fHbp) as a key antigen (21–23). fHbp is a 27-kDa surface lipoprotein that is expressed by almost all disease-causing isolates of N. meningitidis (24, 25). The protein elicits serum bactericidal activity (SBA), an accepted correlate of protection against meningococcal disease (21, 22, 24). fHbp can be classified into one of three variant groups (V1, V2, or V3) or two subfamilies (A and B) based on its amino acid sequence (22, 26). Immunization with fHbp generally induces cross protection against strains expressing an fHbp within the same variant group, with limited cross protective immunity between variant groups (21).

fHbp promotes survival of N. meningitidis in serum by binding the negative complement regulator complement factor H (CFH) at high affinity (25). CFH consists of 20 complement control protein (CCP) domains joined by short linker sequences, with each domain consisting of approximately 60 amino acids (27–29). CFH inhibits the alternative pathway (AP) of complement by acting as a cofactor for factor I-mediated cleavage of C3b, a key molecule in the complement cascade, and by accelerating the decay of the AP C3 convertase (30–32). These functions are mediated by the first four CCP domains (CFH_1–4_), while other CCP domains have distinct functions, such as binding to heparin and glycosaminoglycans (33, 34). N. meningitidis fHbp binds CCP domains 6 and 7 of CFH (CFH_6–7_) (25, 35), while a further complement protein, CFHR3, also binds to fHbp and acts as a competitive antagonist of CFH (36).

Recent genome analysis of 80 pathogenic and commensal Neisseria strains identified *fhbp* in all N. meningitidis strains examined and homologues of *fhbp* in some commensal species (37). A homologue of *fhbp* was also present in isolates of N. gonorrhoeae, although the protein encoded by this gene, Ghfp, does not bind CFH to any significant degree (38, 39). *fhbp* was also found in all isolates of N. cinerea and N. polysaccharea examined (37–39). Of note, *N. cinerea fhbp* genes have significant nucleotide identity with V1 *fhbp*, whereas N. gonorrhoeae and N. polysaccharea harbor homologues of V3 *fhbp* (37, 39).

Several lines of evidence indicate that commensal Neisseria spp. can induce immunity against N. meningitidis. For example, colonization with N. lactamica during early childhood is associated with the development of adaptive immunity against the meningococcus (5, 40, 41). Moreover, individuals colonized with N. lactamica following live challenge develop cross-reactive salivary IgA and serum IgG antibodies and appear to be protected against subsequent acquisition of N. meningitidis (42). Therefore, the presence of antigens, such as fHbp, in commensal Neisseria spp. raises the possibility that implementation of protein-based vaccines containing fHbp could impact the colonization of commensal species in the upper airway.

Here, we investigated whether *fhbp* in the N. cinerea genome encodes a functional protein and examined its roles in binding CFH and complement evasion. We found that N. cinerea expresses fHbp on its surface, where it binds CFH at high affinity and mediates resistance against complement-mediated lysis. Furthermore, we evaluated whether immune responses elicited by Bexsero have significant activity against N. cinerea. We demonstrate that immune responses elicited by Bexsero have SBA against N. cinerea, with fHbp the main target of SBA responses elicited by Bexsero against N. cinerea, indicating that introduction of fHbp-containing vaccines could affect carriage of commensal Neisseria spp.

## RESULTS

### N. cinerea fHbp is predicted to be structurally similar to meningococcal fHbp.

**FIG 1:**
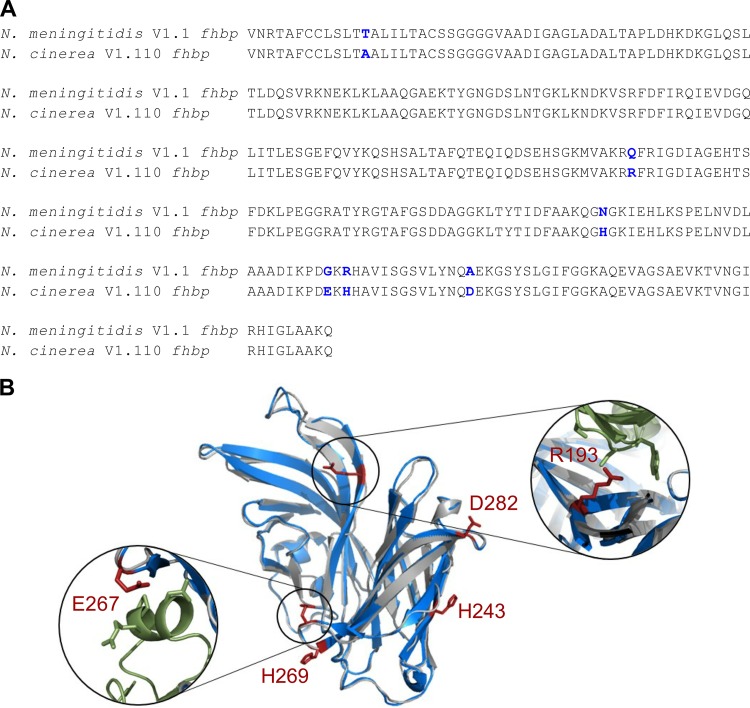
N. cinerea fHbp is predicted to be structurally similar to V1.1 fHbp. (A) Sequence alignment of N. meningitidis V1.1 *fhbp* and N. cinerea V1.110 *fhbp*. The blue residues show sequence differences. (B) Predicted structure of N. cinerea V1.110 fHbp (gray ribbon) generated with the I-TASSER server and V1.1 fHbp (blue ribbon [25]; PDB accession no. 2W80). The figure was drawn using Pymol. Residues in red (shown as ball-and-stick representations) are different in N. cinerea fHbp and V1.1 fHbp. Residues R193 and E267 (red) are in close proximity to the CFH (green ribbon) binding site.

The genome sequences of five N. cinerea isolates, CCUG 346T, CCUG 5746, CCUG 25879, CCUG 27178A, and CCUG 53043, from the PubMLST Neisseria BIGSdb database (http://pubmlst.org/neisseria/) were interrogated for the presence of *fhbp*. Previously annotated *fhbp* sequences were verified by BLAST using the V1.1 *fhbp* nucleotide sequence as the search query. All the isolates contain *fhbp* with a predicted signal peptide, a conserved lipobox, and a variable linker sequence (see Fig. S1 in the supplemental material), with a high degree of nucleotide sequence identity (∼95%) with V1.1 *fhbp* from N. meningitidis, consistent with previous findings (37). Furthermore, N. cinerea CCUG 346T *fhbp* is identical to the meningococcal *fhbp* peptide variant V1.110 (http://pubmlst.org/neisseria/). We further characterized *fhbp* from N. cinerea using CCUG 346T, as the strain has previously been shown to be genetically tractable (43). The predicted amino acid sequence of fHbp from N. cinerea CCUG 346T has six amino acid differences from V1.1 fHbp (Fig. 1A). A structural model of N. cinerea fHbp was generated using V1.1 fHbp as the threading template (25; Protein Data Bank [PDB] accession no. 2W80) (Fig. 1B). One different amino acid, A58, is predicted to reside in the leader peptide, while three of the residues unique to N. cinerea fHbp (H243, H269, and D282) are in the C-terminal barrel of fHbp, distant from the CFH-fHbp interface (44). Residues R193 and E267 in N. cinerea fHbp reside at the CFH-fHbp interface in close proximity (i.e., within 4 Å) to S366 and N338 of CFH, respectively, which could potentially lead to the formation of additional salt bridges between fHbp and CFH. Alanine substitution for Q193 in meningococcal V1.1 fHbp leads to a 2-fold decrease in CFH binding compared to wild-type V1.1 fHbp, while alanine substitution at position 267 in V2.21 fHbp (E267) showed significantly reduced CFH binding, with no binding of CFH_6–7_ detected (44).

### N. cinerea fHbp binds CFH at high affinity.

**FIG 2:**
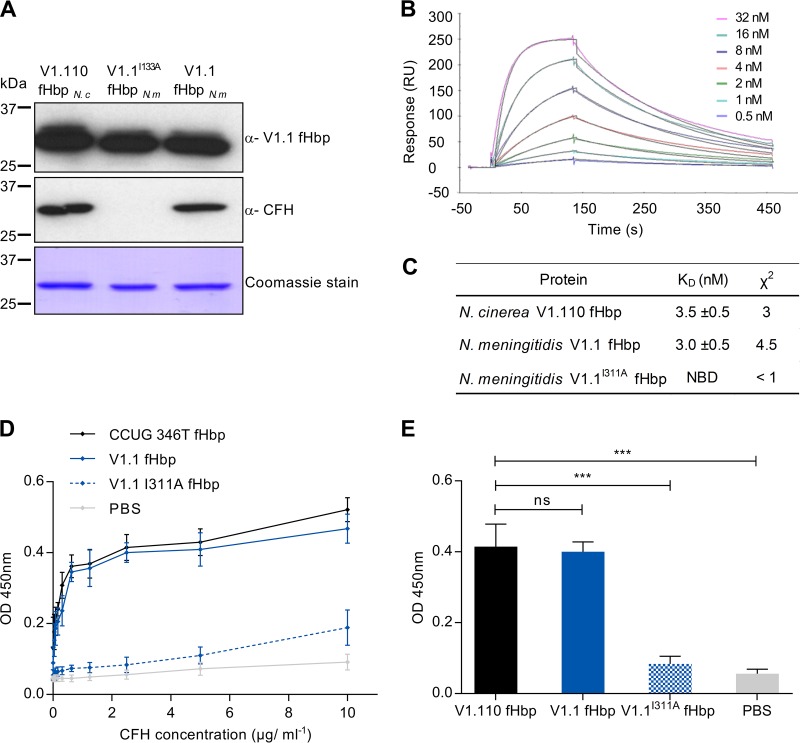
N. cinerea V1.110 fHbp binds CFH at high affinity. (A) Recombinant V1.110 fHbp from N. cinerea (*N c*) CCUG 346T, with V1.1 fHbp, and V1.1^I311A^ fHbp from N. meningitidis (*N m*) were recognized by Western blotting using polyclonal anti-V1.1 fHbp sera. N. cinerea V1.110 fHbp and V1.1 fHbp bound CFH by far-Western analysis with NHS as the source of CFH. Molecular masses are shown, and Coomassie blue staining of proteins is shown as the loading control. (B) SPR analysis of N. cinerea fHbp binding to recombinant CFH_6–7_ (concentrations are indicated) with 1:1 Langmuir fit (black lines). (C) Calculated *K_D_* values for CFH_6–7_ binding to N. cinerea V1.110 fHbp and V1.1 fHbp; NBD, no binding detected. (D) ELISA of N. cinerea fHbp, V1.1 fHbp, and V1.1^I311A^ fHbp binding to full-length CFH. (E) Significance of CFH binding by ELISA analyzed at 5 μg/ml of CFH. The error bars indicate the standard error of the mean (SEM) from three independent experiments, and *P* values (***, *P* < 0.001; ns, *P* > 0.05) were calculated using a two-tailed unpaired *t* test.

As amino acids in N. cinerea and N. meningitidis fHbp proteins differ at the CFH interface, we next evaluated whether N. cinerea fHbp binds CFH (37). N. cinerea fHbp was expressed as a recombinant protein and purified as previously described (44). The protein was recognized by anti-V1.1 fHbp serum by Western blot analysis, as expected (Fig. 2A). Initially, binding of fHbp from N. cinerea and V1.1 fHbp from N. meningitidis to CFH was determined by far-Western analysis using normal human serum (NHS) as the source of CFH. V1.1^I311A^ fHbp was used a negative control, as it has significantly impaired binding to CFH (44). The results of far-Western analysis demonstrated that N. cinerea fHbp and wild-type V1.1 fHbp bind CFH, while V1.1^I311A^ fHbp has no detectable CFH binding, as previously described (44) (Fig. 2A).

To further characterize this interaction, we determined the affinity of N. cinerea fHbp for CFH_6–7_ by surface plasmon resonance (SPR). N. cinerea fHbp binds to CFH_6–7_ with an affinity similar to that of V1.1 fHbp, with equilibrium binding constants (*K_D_*s) of 3.5 ± 0.5 nM and 3.0 ± 0.5 nM, respectively (Fig. 2B and C), indicating that the amino acid substitutions in N. cinerea V1.110 fHbp do not affect its ability to bind CFH. We also determined the levels of CFH binding to N. cinerea fHbp and V1.1 fHbp by enzyme-linked immunosorbent assay (ELISA) using full-length CFH (Fig. 2D and E). The ELISA data were consistent with results from both far-Western and SPR analyses, with no significant difference in CFH binding to N. cinerea fHbp and V1.1 fHbp over a range of concentrations (*P* > 0.05 by two-tailed *t* test) (Fig. 2E). Taken together, these results demonstrate that N. cinerea fHbp binds CFH and that this interaction is mediated by CFH_6–7_ of CFH.

### N. cinerea isolates express fHbp on the bacterial surface.

**FIG 3:**
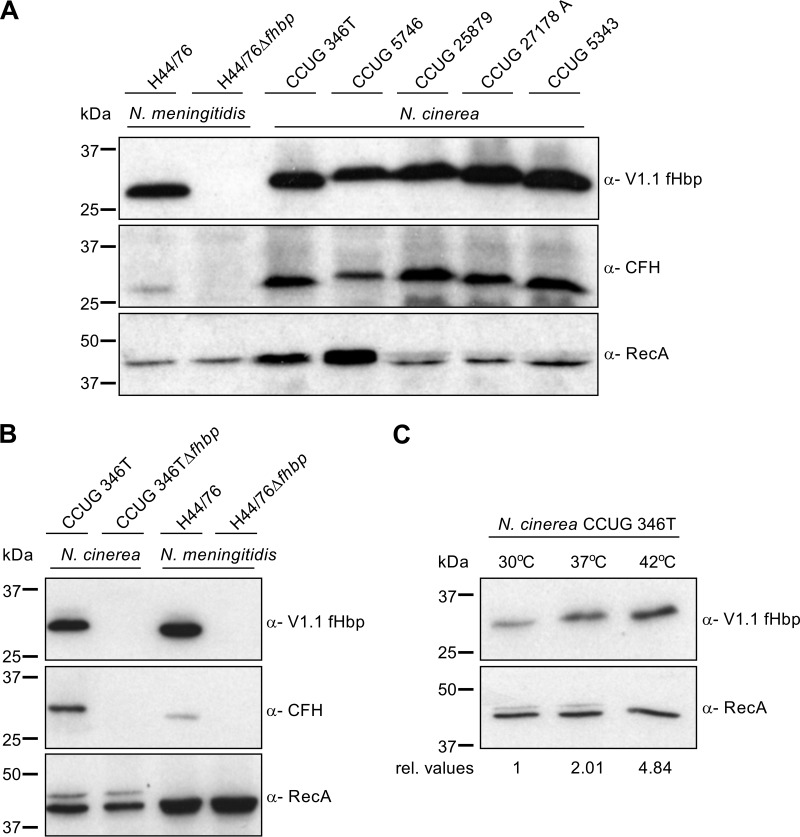
Functional fHbp was expressed by all N. cinerea strains examined. (A) Western blot analysis of fHbp expression using anti-V1.1 fHbp sera against N. cinerea strains and N. meningitidis H44/76 and H44/76Δ*fhbp*. Far-Western analysis demonstrated that fHbps from different N. cinerea isolates bound CFH. RecA was used as a loading control. (B) Western and far-Western analyses demonstrating that binding of CFH to N. cinerea is dependent on fHbp expression. (C) Temperature-dependent regulation of fHbp expression by N. cinerea, examined by Western blot analysis using anti-V1.1 fHbp sera. RecA, loading control, with relative fHbp expression levels (rel. values) shown.

Next, the expression of fHbp by other N. cinerea isolates was analyzed using polyclonal mouse serum raised against V1.1 fHbp, which cross-reacts with N. cinerea fHbp from CCUG 346T (Fig. 2A). A band of approximately 27 kDa corresponding to the molecular mass of fHbp was detected in lysates of all five N. cinerea isolates by Western blotting (Fig. 3A). Moreover, five N. cinerea isolates were analyzed for the ability to bind full-length CFH present in NHS; N. meningitidis H44/76 and H44/76Δ*fhbp* were used as positive and negative controls, respectively. Far-Western analysis detected a band corresponding to fHbp in whole-cell lysates of N. cinerea isolates and the positive control, H44/76; no CFH binding to N. meningitidis H44/76Δ*fhbp* was detected, as previously described (39) (Fig. 3A). We also sought to establish whether CFH binding to N. cinerea is dependent on fHbp. Therefore, we constructed an *fhbp* mutant of N. cinerea CCUG 346T by insertional inactivation of the gene with a kanamycin resistance cassette. Analysis of the wild-type N. cinerea and the Δ*fhbp* strain demonstrated that the Δ*fhbp* mutant did not express fHbp, as expected, while far-Western blot analysis demonstrated that CFH binding to N. cinerea is dependent on fHbp (Fig. 3B).

Our previous work demonstrated that N. meningitidis fHbp is subject to thermal regulation, with higher levels of fHbp produced by strains grown at higher temperatures (i.e., 37°C and 42°C compared with 30°C) (45, 46). Therefore, we investigated whether N. cinerea fHbp is also thermoregulated. N. cinerea CCUG 346T was harvested following growth of the bacteria in brain heart infusion (BHI) broth to mid-log phase at 30°C, 37°C, or 42°C; the relative fHbp expression levels were quantified by Western blot analysis (Fig. 3C). The results demonstrated that increasing temperature is associated with elevated fHbp expression in N. cinerea, similar to the meningococcus (*P* = 0.0382; one-way analysis of variance [ANOVA] for 30°C versus 42°C) (Fig. 3C). Inspection of sequences corresponding to the 5′ untranslated region (UTR) of *fhbp* from N. cinerea CCUG 346T indicated that there is a single base change (C-U) in the fumarate-nitrate reduction regulator (FNR) box, but the temperature-regulatory domains identified in N. meningitidis MC58 (46) are conserved in N. cinerea CCUG 346T (see Fig. S2 in the supplemental material). Two putative α-ribosome binding sites (α-RBS) in the open reading frame (ORF) of fHbp of N. meningitidis MC58 have been shown to determine temperature regulation of fHbp (46), and N. cinerea CCUG 346T retains an identical sequence for α-RBS1 (CUGCCU) (see Fig. S2 in the supplemental material) but has a C-U substitution in the second α-RBS.

**FIG 4:**
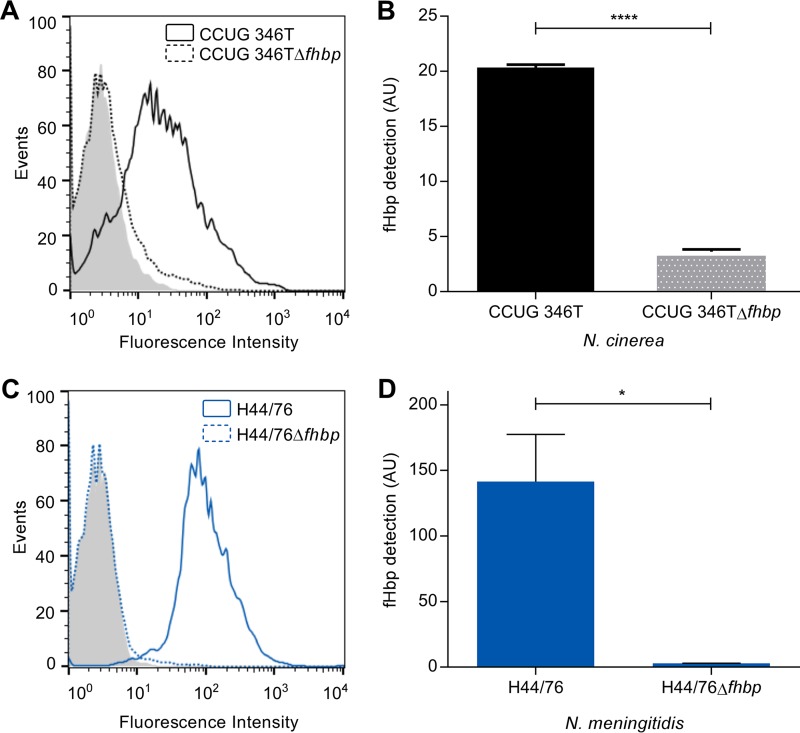
N. cinerea fHbp is present on the bacterial surface. Flow cytometry analysis with anti-V1.1 fHbp serum demonstrated detection of fHbp on the surfaces of N. cinerea (solid black line) (A and B) and N. meningitidis (solid blue line) (C and D). The error bars indicate the standard error of the mean (SEM) from three independent experiments. Significance was calculated using a two-tailed unpaired *t* test; ****, *P* < 0.0001; *, *P* < 0.05.

To analyze surface expression of fHbp in N. cinerea, we performed flow cytometry analysis using anti-V1.1 fHbp serum to detect fHbp on the surfaces of N. cinerea CCUG 346T and CCUG 346TΔ*fhbp* (Fig. 4A and B); N. meningitidis H44/76 and H44/76Δ*fhbp* were included as positive and negative controls, respectively (Fig. 4C and D). The results demonstrated that fHbp is detected on the surface of N. cinerea CCUG 346T, as well as N. meningitidis H44/76; fHbp was not detected on either of the *fhbp* mutants (two-tailed *t* test; *P* < 0.0001 and *P* = 0.0183 compared with the respective wild-type strains).

### N. cinerea fHbp promotes complement resistance.

**FIG 5:**
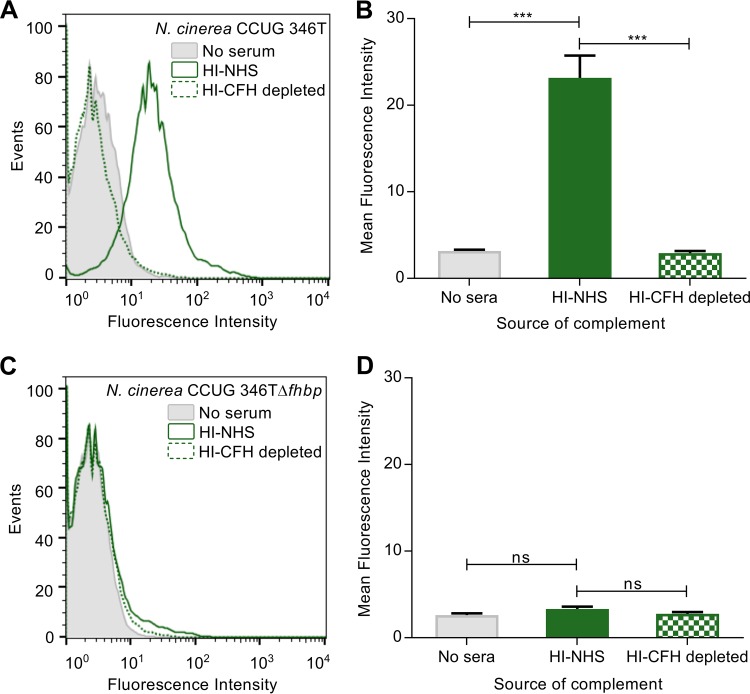
Surface-bound CFH contributes to complement resistance of N. cinerea in an fHbp-dependent manner. Shown is flow cytometry analysis of CFH binding to N. cinerea CCUG 346T (A and B) and N. cinerea CCUG 346TΔ*fhbp* (C and D). The shaded areas indicate results from bacteria incubated with PBS, the solid green lines show bacteria incubated in HI-NHS, and the dashed green lines show bacteria incubated in HI-CFH-depleted sera. The data are presented as the mean fluorescence intensity. The error bars indicate the standard error of the mean (SEM) from three independent experiments, and the *P* values were calculated using a two-tailed unpaired *t* test; ***, *P* < 0.001; ns, *P* > 0.05.

Next, we investigated whether fHbp can recruit CFH to the surface of N. cinerea. N. cinerea CCUG 346T and CCUG 346TΔ*fhbp* were incubated in heat-inactivated (HI) NHS as a source of CFH, and CFH was detected by flow cytometry with an α-CFH monoclonal antibody (MAb), OX24 (Fig. 5A and B) (47); binding was quantified as the geometric mean fluorescence intensity. Bacteria incubated in phosphate-buffered saline (PBS) or in CFH-depleted serum (HI-CFH depleted) were used as controls. Following incubation with HI-NHS, CFH was detected on the surface of N. cinerea (Fig. 5A and B), while no CFH binding was found either after incubating wild-type bacteria with HI-CFH-depleted serum or PBS or after incubating the *fhbp* mutant in HI-NHS (two-tailed *t* test; *P* = 0.0002) (Fig. 5A to D), demonstrating that CFH binds to the surface of N. cinerea in an fHbp-dependent manner.

**FIG 6:**
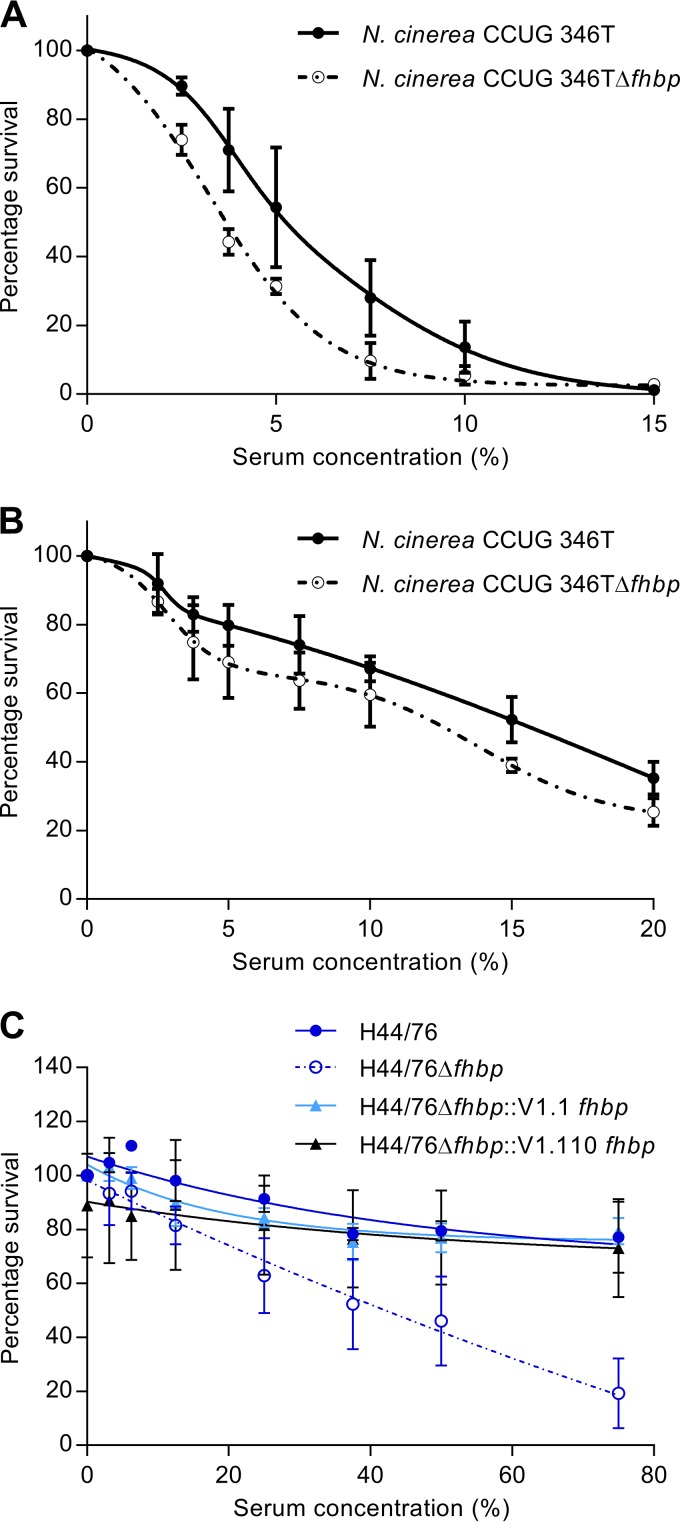
N. cinerea fHbp contributes to complement resistance. (A and B) Sensitivity of N. cinerea and N. cinerea CCUG 346TΔ*fhbp* to complement-mediated lysis in the presence of NHS (A) and in the presence of the AP of complement only (B). (C) Survival in human serum of N. meningitidis H44/76, H44/76Δ*fhbp*, and H44/76Δ*fhbp* complemented with either V1.1 fHbp or N. cinerea V1.110 fHbp. The error bars indicate the standard error of the mean (SEM) from three independent experiments.

Next, we examined the functional consequences of CFH binding to N. cinerea by performing serum survival assays. N. cinerea CCUG 346T and CCUG 346TΔ*fhbp* were incubated in NHS (concentrations ranging from 0 to 20%) for 30 min, and the percent survival was calculated by comparing the number of CFU recovered after incubation in serum with the number of CFU recovered after incubation in PBS. N. cinerea CCUG 346T was significantly more resistant to complement-mediated lysis than CCUG 346TΔ*fhbp* over a range of serum concentrations (Fig. 6A). For example, there was 54% and 31% survival for CCUG 346T and CCUG 346TΔ*fhbp*, respectively, in 5% NHS (two-tailed *t* test; *P* = 0.0161). Furthermore, we examined whether these differences in survival were observed in the presence of only the AP of complement by inhibiting the activities of the classical and lectin pathways by the addition of 10 mM EGTA and 5 mM MgCl_2_ to sera (48). N. cinerea CCUG 346T still demonstrated a trend toward enhanced serum resistance compared to CCUG 346TΔ*fhbp* when only the AP was active in a 15% serum concentration, although this did not reach statistical significance (two-tailed *t* test; *P* = 0.127) (52.28% and 38.94% survival for CCUG 346T and CCUG 346TΔ*fhbp*, respectively) (Fig. 6B). Furthermore, we analyzed whether an *N. meningitidis fhbp* mutant, H44/76Δ*fhbp*, could be complemented with N. cinerea fHbp (V1.110). N. meningitidis H44/76, H44/76Δ*fhbp*, and H44/76Δ*fhbp* complemented with either meningococcal V1.1 fHbp or N. cinerea fHbp were incubated in NHS (concentrations ranging from 0 to 75%) for 30 min, and the percent survival was calculated. Both complemented strains, H44/76Δ*fhbp*::V1.1*fhbp* and H44/76Δ*fhbp*::V1.110*fhbp*, were significantly more resistant to NHS than H44/76Δ*fhbp* (one-way ANOVA; *P* = 0.0023 and *P* = 0.0046, respectively), and no significant difference in survival was observed between the complemented strains and H44/76 at 75% NHS (Fig. 6C). Taken together, these results demonstrate that N. cinerea fHbp contributes to the evasion of killing by the complement system.

### fHbp-containing vaccines elicit SBA against N. cinerea.

**FIG 7:**
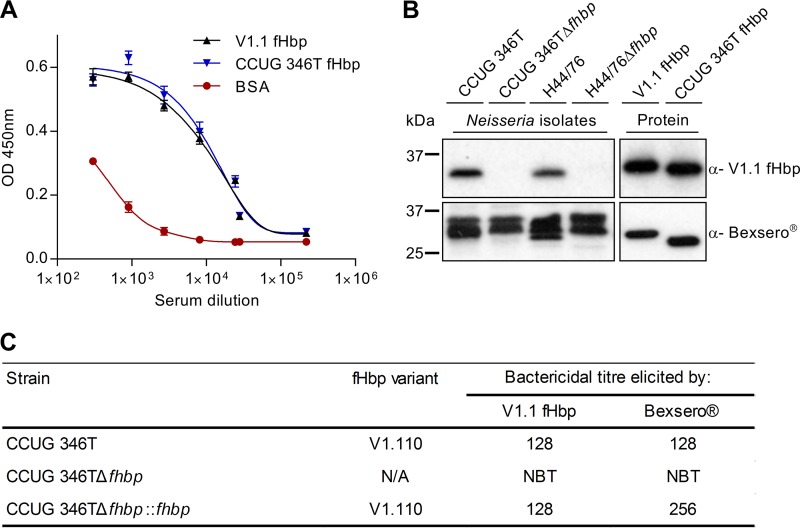
Immunization with Bexsero elicits SBA against N. cinerea. (A) ELISA analysis of anti-Bexsero mouse sera (3-fold dilutions from 1/300) against recombinant fHbp; BSA was used as a control. The error bars indicate the standard error of the mean (SEM) from three independent experiments. (B) fHbp (∼27 kDa) is recognized by sera from mice immunized with V1.1 fHbp or Bexsero as determined by Western blot analysis. The higher-molecular-mass band observed when whole-cell extracts were probed with sera from mice immunized with Bexsero was likely due to responses against other proteins in the vaccine. (C) Serum bactericidal titers of pooled sera from mice immunized with recombinant V1.1 fHbp or Bexsero against N. cinerea strains; NBT, no bactericidal titer.

Finally we examined whether the licensed meningococcal vaccine, Bexsero, which contains meningococcal fHbp, elicits immune responses against N. cinerea. Mice were immunized with Bexsero or an equivalent amount (20 μg) of V1.1 fHbp; sera from mice immunized with adjuvant alone were used as a negative control. Initially, we used ELISA to measure the ability of antibodies in pooled immune sera to recognize V1.1 fHbp and N. cinerea fHbp. Interestingly, there was no significant difference in the recognition of V1.1 fHbp and N. cinerea fHbp by antibodies in sera from mice immunized with Bexsero or V1.1 fHbp (Fig. 7A; data from mice immunized with fHbp V1.1 not shown) (*P* > 0.05 by two-tailed *t* test). Next, the reactivity of sera was examined by Western blotting of whole-cell lysates from N. cinerea CCUG 346T and N. meningitidis H44/76 (Fig. 7B). Sera from mice immunized with V1.1 fHbp detected a band of the size expected for fHbp by Western blot analysis of lysates of wild-type strains, while no protein was detected in the Δ*fhbp* mutants, as previously observed for polyclonal sera against V1.1 fHbp (see Fig. S3A in the supplemental material) (44). As expected, sera from mice immunized with Bexsero detected multiple protein bands in whole-cell lysates (see Fig. S3B) due to the complex mixture of the vaccine (23), although a band corresponding to the molecular mass of fHbp was detected in lysates of N. cinerea CCUG 346T, but not from the Δ*fhbp* mutant. Sera from mice immunized with either recombinant fHbp or Bexsero also recognized recombinant V1.1 and N. cinerea V1.110 fHbp (Fig. 7B).

Next, the potential impact of immune responses raised against Bexsero on N. cinerea was examined by measuring SBA. Immunization with V1.1 fHbp or Bexsero elicited SBA titers of 128 against N. cinerea CCUG 346T; SBA titers of ≥8 are correlated with protection against serogroup B meningococcal disease (Fig. 7C) (49). Of note, sera from mice immunized with Bexsero had a higher SBA titer (256) against N. cinerea CCUG 346T complemented with N. cinerea fHbp under an IPTG (isopropyl-β-d-thiogalactopyranoside)-inducible promoter than wild-type bacteria, consistent with higher expression of fHbp by the complemented strain (not shown). Furthermore, no SBA was detected against N. cinerea CCUG 346TΔ*fhbp* with either V1.1 fHbp or Bexsero, demonstrating that SBA elicited by Bexsero against N. cinerea is mediated by fHbp. In conclusion, immune responses elicited by Bexsero recognize N. cinerea fHbp and have significant SBA.

## DISCUSSION

fHbp is a key virulence factor for the meningococcus and binds the negative complement regulator CFH, promoting bacterial survival in human serum (25, 50, 51). The importance of fHbp as a vaccine antigen is evident from its inclusion in two recently licensed serogroup B meningococcal vaccines (21, 22). Here, we show that fHbp expression is not restricted to pathogenic N. meningitidis but that the antigen is also expressed by the commensal species N. cinerea, in which fHbp is surface located and binds CFH at high affinity. Recruitment of CFH by N. cinerea promotes its survival in human serum complement, while serum raised against Bexsero has SBA against N. cinerea expressing fHbp.

Previous work has analyzed the expression and function of fHbp in pathogenic Neisseria spp. (39, 50, 51). fHbp is a highly variable protein that can be grouped into three distinct variants based on its amino acid sequence (26). Using SPR, we found that N. cinerea fHbp binds CFH in the nanomolar range, similar to the affinity of CFH for V1.1 fHbp from N. meningitidis, indicating that high-affinity binding of CFH to the surface of the bacterium is not only involved during survival in the bloodstream, but likely aids colonization and persistence in the human nasopharynx. In contrast, N. gonorrhoeae, which resides in the genitourinary tract, expresses a nonfunctional fHbp called Ghfp (39) and instead recruits CFH via an abundant surface porin, PorB.1 (52, 53). A frameshift mutation in the nucleotide sequence at position 40 of *ghfp* disrupts the lipobox, resulting in the loss of surface localization of Ghfp (39), and sequence changes in *ghfp* result in a protein that is unable to bind CFH (39). The distinct mechanisms by which N. meningitidis and N. cinerea recruit CFH compared with N. gonorrhoeae are likely to reflect differences in concentrations of CFH and the levels of other complement proteins at the sites they occupy in the body. Complement proteins, including C3b, may be derived from exudates on the nasopharyngeal mucosa from the subepithelial microvasculature (54, 55). Levels of C3 in nasopharyngeal secretions are estimated to be approximately 6.6% of serum C3 levels (range, 4.2 to 20.2%), indicating that there is a significant level of complement in the nasopharynx (56). Although CFH could not be detected on respiratory epithelial cells by immunohistochemistry (57), levels of the complement regulator in saliva range from 0.007 to 0.059 μg/ml (0.04 to 0.38 nM) (58, 59), which is significantly lower than in serum. There are few studies on CFH in the genital tract, although low levels of CFH have been detected in urothelial cells, which also can produce C3 (57). High-affinity interactions with CFH might be required at mucosal surfaces due to low availability of this important complement regulator (58, 59). Furthermore, levels of CFH and other complement proteins may change during inflammation, when CFH recruitment might provide an advantage for commensal and pathogenic bacteria in avoiding exudates of serum components and putatively could aid N. cinerea colonization of the nasopharynx (56, 60, 61).

Binding of CFH is important for nasopharyngeal colonization by other bacteria, including Streptococcus pneumoniae (62), by mediating interactions with respiratory epithelial cells. Binding of CFH to PspC on the pneumococcus significantly increases its attachment to human epithelial and endothelial cells by acting as a molecular bridge between the bacteria and host cells (63). Moreover, the RGD motif in CCP_4_ of CFH can recognize integrins on cells to induce uptake of the pneumococcus (63, 64). A similar role for CFH acting as a molecular bridge has not been described for N. meningitidis or N. cinerea as yet and merits further investigation, as similar mechanisms might influence N. meningitidis and N. cinerea colonization of the human nasopharynx.

Although N. cinerea expresses *fhbp*, other, closely related commensal species, such as N. lactamica and N. polysaccharea, do not express fHbp. The majority of N. polysaccharea isolates have a frameshift in the ORF, potentially producing a truncated protein, while *fhbp* has not been identified in the N. lactamica genome, indicating that fHbp is not essential for successful colonization of the human nasopharynx by Neisseria spp.

Previous work has suggested that NspA and PorB2 of N. meningitidis can bind CFH, although these proteins have little effect in wild-type strains and specific binding affinities are not known (65, 66). Although N. cinerea CCUG 346T carries a gene encoding PorB2, we found no evidence by Western blotting or flow cytometric analysis that molecules other than fHbp are responsible for N. cinerea binding to CFH. Of note, N. cinerea CCUG 346T is not predicted to harbor a functional *nspA* gene. Other functional roles that could enhance bacterial survival have also been attributed to meningococcal fHbp, including protecting against the cationic antimicrobial peptide LL-37 (67). LL-37 is a cathelicidin that is expressed by epithelial cells in the nasopharynx (68, 69). Furthermore, both fHbp and Ghfp have been postulated to act as siderophore receptors and to recruit enterobactin and so could contribute to iron scavenging on mucosal surfaces (70).

Previously, we demonstrated that meningococcal fHbp levels are elevated with increasing temperatures, i.e., from 30°C to 37 and 42°C (45, 46). Thermoregulation of meningococcal fHbp is mediated by two putative α-RBS within the ORF of *fhbp*; at lower temperatures, the transcript forms a stem-loop structure including the RBS, thus reducing fHbp expression (46, 71). Sequence analysis of N. cinerea CCUG 346T indicated that the 5′ UTR upstream of *fhbp*, including the monocistronic promoter, is conserved compared with N. meningitidis MC58 and includes the RBS and one repeat of the α-RBS within the *fhbp* ORF (see Fig. S2 in the supplemental material). The second α-RBS has a single residue change, C-U, although N. cinerea fHbp is still subject to temperature regulation (Fig. 3C). Temperature is also an indicator of local inflammation, which could expose the bacterium to host immune effectors. Therefore, upregulation of fHbp could provide N. cinerea with a selective advantage over other commensal Neisseria spp. that do not expresses the antigen (72, 73).

Conjugate vaccines against N. meningitidis have been highly successful at reducing carriage of the pathogen but do not affect commensal Neisseria spp., as they target specific capsular polysaccharides that are not expressed by commensal species (74, 75). Limited data are currently available on the impact of Bexsero and other protein-based vaccines on meningococcal carriage (76, 77). As IgG and complement are present on mucosal surfaces (54, 78, 79), it is possible that immune responses against fHbp could affect the nasopharyngeal carriage of bacteria expressing fHbp. We found that immunization of mice with Bexsero elicited SBA against N. cinerea, with titers similar to those found in mice immunized with fHbp alone. Furthermore, there was no SBA against N. cinerea lacking fHbp, indicating that fHbp is largely responsible for the SBA seen with Bexsero. With regard to other protein antigens found in Bexsero, genes encoding GNA2091 and GNA1030 are present in all N. cinerea isolates, whereas only fragments of *nadA* are found in some isolates (37). NHBA and PorA have not been identified in any N. cinerea isolate analyzed to date. However, as N. cinerea lacks a capsule, it may be more susceptible than N. meningitidis to immune responses elicited by Bexsero against surface proteins. Therefore, it will be important to evaluate the impact of Bexsero on the carriage of commensal Neisseria, as carriage of nonpathogenic species can induce cross protective immunity (5, 42, 80).

In summary, we have shown that N. cinerea expresses functional fHbp on its surface, where it contributes to complement evasion. Moreover, Bexsero elicits bactericidal antibodies against N. cinerea, with SBA responses similar to immunization with fHbp alone. Therefore, implementation of vaccines containing fHbp has the potential to affect the commensal nasopharyngeal flora, and the impact of multicomponent vaccines should be assessed in future challenge studies.

## MATERIALS AND METHODS

### Sequence analysis and model of N. cinerea fHbp.

*N. cinerea fhbp* sequences were extracted from previously annotated whole-genome sequences available in the PubMLST BIGSdb database (http://pubmlst.org/neisseria/). Alignments of the predicted N. cinerea fHbp sequence against the V1.1 fHbp were generated by Clustal OMEGA (http://www.ebi.ac.uk/Tools/msa/clustalo/); residues were numbered according to V1.1 fhbp. A model of N. cinerea fHbp was generated using the iTASSER server (81) with V1.1 fHbp (25; PDB accession no. 2W80). The resulting model is illustrated in PyMol (https://www.pymol.org/) and has high confidence, with a C score of 1.68.

### Bacterial strains and growth.

The strains used in this study are listed in Table 1. Neisseria species were grown on BHI agar (1.5% [wt/vol]; Oxoid) supplemented with 5% defibrinated horse blood. Bacteria grown on solid media were incubated overnight at 37°C in the presence of 5% CO_2_. Liquid cultures in BHI broth were inoculated with 10^9^ CFU per 10 ml and grown with shaking at 180 rpm at the specified temperatures to an *A*_600_ of ∼0.5 unless otherwise indicated. Antibiotics were added to media at the following concentrations: kanamycin, 100 μg ml^−1^, and erythromycin, 10 μg ml^−1^. Escherichia coli was grown in Luria-Bertani (LB) broth or on LB agar. Overnight liquid cultures were grown at 37°C with shaking at 180 rpm in 5 ml of medium inoculated from a single colony. The bacteria were diluted 1 in 100 the next morning into larger volumes for protein expression. Antibiotics were added at the following concentrations for E. coli: kanamycin, 50 μg ml^−1^, and carbenicillin, 100 μg ml^−1^.

**TABLE 1:** N. cinerea and N. meningitidis strains used in this study

Strain	fHbp variant	Yr identified	Reference
N. cinerea			
CCUG 346T	V1.110	Unknown	7
CCUG 346TΔ*fHbp*			This study
CCUG 346TΔ*fHbp*::*fhbp*	V1.110		This study
CCUG 25879	V1.564	1989	7
CCUG 27178 A	V1.110	1983	7
CCUG 53043	V1.534	2006	85
CCUG 5746	V1.563	1977	7
N. meningitidis			
H44/76	V1.1	1976	86
H44/76Δ*fhbp*			39
H44/76Δ*fhbp*::V1.1 *fhbp*	V1.1		39
H44/76Δ*fhbp*::V1.110 *fhbp*	V1.110		This study

### Generation of plasmids and protein purification.

Genomic DNA was isolated using a Wizard Genomic DNA purification kit (Promega) according to the manufacturer's instructions. *fhbp* was amplified from N. cinerea genomic DNA using primers F_*fhbp*_ (5′-GCCATATGATGGCCGCCGACAT-3′; NdeI site underlined) and R_*fhbp*_ (5′-GCCTCGAGTTGCTTGGCGGCAAGGCCGATAT-3′; XhoI site underlined). The product was ligated into pET-21b (Table 2), resulting in a construct for expression of fHbp with a C-terminal His tag under an inducible promoter. fHbp expression was performed as described previously (44).

**TABLE 2:** E. coli BL21(DE3)Lys plasmids

Plasmid	Insert	Modification	Source
pET21b	V1.1 *fhbp*		44
pET21b	V1.1 *fhbp*	I311A	44
pET21b	V1.110 *fhbp*		This study

In brief, for protein expression, E. coli BL21(D3) (Agilent) containing fHbp expression constructs was grown in liquid medium to an *A*_600_ of 0.4 to 0.8; then, IPTG was added to a final concentration of 1 mM. After 4 h, the bacteria were harvested by centrifugation at 5,000 × *g* for 30 min at 4°C prior to cell lysis in an EmulsiFlex-C5 homogenizer (Avestin) at 15,000 lb/in^2^. The lysates were centrifuged at 50,000 × *g* for 30 min at 4°C, and recombinant fHbp was purified by affinity chromatography using a HisTrap column (GE Healthcare) and eluted with 200 mM imidazole. Further purification was performed with an AKTA purifier (GE Healthcare) by anion-exchange chromatography (HiTrapQ HP column; GE Healthcare). Protein concentrations were estimated using a Nanodrop 2000c spectrophotometer (Thermo Scientific).

### SDS-PAGE and Western blot analysis.

Recombinant protein (10 μg) and whole-cell extracts were separated on 12% or 14% polyacrylamide gels prior to transfer to Immobilon P polyvinylidene difluoride (PVDF) membranes (Millipore, USA) using the Trans-Blot SD semidry transfer system (Bio-Rad, USA) or staining for 10 min with Coomassie blue. For whole-cell extracts, N. meningitidis and N. cinerea were grown on solid medium overnight and resuspended in PBS. The number of bacteria was determined by measuring the DNA concentration by the *A*_260_ of 20 μl of the bacterial suspension in 980 μl of lysis buffer (0.1 M NaOH, 1% SDS); 10^9^ CFU was mixed with an equal volume of 2× SDS-PAGE loading buffer (100 mM Tris-HCl, pH 6.8, 20 μM β-mercaptoethanol, 4% SDS, 0.2% bromophenol blue, 20% glycerol) and boiled for 10 min. For Western blot analysis, membranes were blocked in 0.05% (wt/vol) dry milk-PBS with 0.05% (vol/vol) Tween 20 overnight and then incubated with primary antibodies or with NHS as a source of CFH. Primary antibodies were used at the following concentrations: anti-V1.1 fHbp polyclonal sera, 1:1,000 (44); anti-RecA, 1:5,000 (Abcam, Cambridge); anti-CFH, 1:10,000 (Millipore). Horseradish peroxidase (HRP)-conjugated secondary antibodies, goat anti-mouse HRP (Dako, United Kingdom), rabbit anti-goat HRP (Dako, United Kingdom), and goat anti-rabbit HRP (Santa Cruz Biotechnology, Germany), were used at a final concentration of 1:10,000. Membranes were washed three times in PBS with 0.05% (vol/vol) Tween 20, and binding was detected using an ECL Western blotting detection kit (Amersham, USA).

To examine the effect of temperature on fHbp expression, N. cinerea was grown on solid medium overnight at 37°C prior to inoculating BHI broth (10 ml) with 10^9^ bacteria. Cultures were grown to mid-log phase (*A*_600_, ∼0.5) at 30°C, 37°C, or 42°C. Bacteria were collected by centrifugation (14,000 × *g* for 5 min at room temperature) and then resuspended in 100 μl sample buffer prior to analysis by Western blotting. Relative expression values were calculated by measuring band intensities with ImageJ software; normalized to the signal for RecA, the loading control; and shown as a ratio relative to bacteria grown at 30°C.

### Surface plasmon resonance and ELISA.

SPR was performed using a BiaCore 3000 instrument (GE Healthcare). The purified proteins were immobilized on a CM5 sensor chip to approximately 800 reaction units (RU) by amine coupling (GE Healthcare). Unreacted groups on the chip were inactivated with ethanolamine. HSB-EB (HBS buffer; 0.01 M HEPES, 150 mM NaCl, 3 mM EDTA, pH 7.4) was used throughout. Increasing concentrations of CFH_6–7_, prepared as described previously (82) (0.5 nM to 32 nM), were injected over the chip at a flow rate of 40 μl/min using the KINJECT command with a dissociation time of 300 s. *K_D_* values were calculated and analyzed with BIAevaluation software. Kinetic data were referenced against both a blank cell and subtraction of a blank injection (HSB-EB).

To determine CFH binding by ELISA, 96-well plates (F96 MaxiSorp; Nunc) were coated with recombinant fHbp (3 μg/ml; 50 μl per well) overnight at room temperature prior to blocking with 3% bovine serum albumin (BSA) in PBS with 0.05% Tween 20. The plates were incubated with full-length human CFH (Sigma) in 2-fold dilutions from 10 μg/ml, and binding was detected with anti-CFH antibody (Millipore) and HRP-conjugated goat anti-mouse polyclonal antibody (Dako). CFH binding was visualized with 3,3′,5,5′-tetramethylbenzidine (TMB) substrate reagent (Roche) and 2 N sulfuric acid stop solution (Roche) according to the manufacturer's instructions, and the *A*_450_ was measured (SpectraMax M5; Molecular Devices). Statistical significance was tested using an unpaired Student *t* test (GraphPad Prism v.6.0) to compare means and standard deviations (SD), with a *P* value of <0.05 as the cutoff for significance. To analyze the reactivity of sera from mice immunized with Bexsero, ELISA plates were coated with V1.1 fHbp, V1.110 fHbp, or BSA (2.5 μg/ml; 50 μl per well) prior to incubation with sera; antibody binding was detected as described above. Pooled mouse sera were used as a primary antibody with 3-fold dilutions from a starting dilution of 1:300.

### Generation of *N. cinerea fhbp* mutants and complemented strains.

N. cinerea strain CCUG 346TΔ*fhbp* was constructed by insertional inactivation of the *fhbp* gene with a cassette encoding kanamycin resistance. *Nc*Δ*fhbp* (forward primer; 5′-GCCATATGGCTGAAAGTCATCAACGAAT-3′ [NdeI site underlined]) and *Nc*Δ*fhbp* (reverse primer; 5′-GCCTCGAGGTGCAATACAAAATGCCGTCCGAACGGTAA-3′ [XhoI site underlined]) were used to amplify *fhbp* and flanking sequences from N. meningitidis strain H44/76Δ*fhbp*. The flanking sequences between N. meningitidis H44/76 and N. cinerea CCUG 346T shared 99.28% nucleic acid sequence identity. N. cinerea CCUG 346 T was then transformed with a linear PCR fragment as described previously (83). The complemented N. cinerea and N. meningitidis strains CCUG 346TΔ*fhbp*::*fhbp* and H44/76Δfhbp::V1.110 were constructed by amplifying *fhbp* from N. cinerea genomic DNA using primers *Nc fhbp* forward (5′-CGGTTAATTAAGGAGTAATTTTTGTGAACC-3′; PacI site underlined) and *Nc fhbp* reverse (5′-CGGTTAATTAATTATTGCTTGGCGGC-3′; PacI site underlined). The PCR products were ligated into pNCC1 for transformation into N. cinerea (43) or PGCC4 for transformation into N. meningitidis (84), which were linearized by ClaI digestion before transforming into N. cinerea or N. meningitidis as described previously (83).

### Flow cytometry.

N. meningitidis and N. cinerea were grown on solid medium overnight and quantified as described above. The bacteria (10^9^ CFU) were fixed in 3% paraformaldehyde for 2 h at room temperature and then washed with PBS. fHbp was detected using anti-V1.1 fHbp serum and goat anti-mouse IgG Alexa Fluor 647-conjugated polyclonal antibody (Molecular Probes, Life Technologies). To evaluate CFH binding, 5 × 10^7^ CFU was resuspended in 20 μl of HI-NHS or CFH-depleted serum (CompTech, USA) for 30 min at room temperature. Binding of CFH was detected with an anti-CFH MAb (OX24) and goat anti-mouse IgG Alexa Fluor 647-conjugated polyclonal antibody (Molecular Probes, Life Technologies) (47). Samples were analyzed using a FACSCalibur (BD Biosciences), and at least 10^4^ events were recorded; the results were analyzed by calculating the geometric mean fluorescence intensity in FlowJo vX software (Tree Star). Statistical significance was tested using an unpaired Student *t* test (GraphPad Prism v.6.0).

### Serum sensitivity assays.

NHS was obtained by collecting venous blood and allowing it to coagulate at room temperature for 60 min before centrifuging it at 3,000 × *g* for 20 min at 4°C prior to storage at −80°C. The NHS was heat inactivated (HI-NHS) at 56°C for 30 min to inactivate complement. N. cinerea and N. meningitidis were grown overnight on BHI agar, and then 10^4^ CFU was incubated in dilutions of NHS in PBS (0 to 20% [vol/vol] for N. cinerea; 0 to 75% [vol/vol] for N. meningitidis) for 30 min at 37°C in the presence of CO_2_. To analyze the effect of the AP on bacterial survival, NHS was preincubated with 5 mM MgCl_2_-10 mM EGTA to inhibit the classical and lectin pathways (48). Bacterial survival was determined by plating onto BHI agar in triplicate. The percent survival was calculated by comparing bacterial recovery in serum with recovery from samples containing no serum. Statistical significance was tested using an unpaired Student *t* test or one-way ANOVA (GraphPad Prism v.6.0).

### Generation of immune sera and SBA.

Eight female BALB/c mice (6 to 8 weeks old; Charles River, Margate, UK) were immunized with recombinant fHbp (20 μg) adsorbed to aluminum hydroxide [final composition, 0.5 mg/ml Al(OH)_3_, 10 mM histidine-HCl] by mixing overnight at 4°C or with Bexsero (total protein, 20 μg). Antigens were given by the intraperitoneal route on days 0, 21, and 35, and sera were collected on day 49 by terminal anesthesia and cardiac puncture. All animal experiments were carried out under protocols reviewed and approved by the Home Office, United Kingdom, under license number PPL 30/3194.

To measure SBA, N. cinerea was grown overnight on BHI agar prior to replating onto solid medium and was grown for a further 5 h at 37°C in 5% CO_2_. A total of 5 × 10^4^ CFU/ml of N. cinerea was mixed with an equal volume of baby rabbit complement (Cedarlane) diluted 1:10 in SBA buffer (Dulbecco's PBS containing 0.1% [wt/vol] glucose, 1 mM CaCl_2_, 0.5 mM MgCl_2_). Sera from individual mice were pooled and heat inactivated for 1 h at 56°C prior to being added to wells in 2-fold dilutions starting at 1:8. Control wells contained either no serum or no complement. Following incubation for 1 h at 37°C, 10 μl from each well was plated onto BHI agar in triplicate, and the number of surviving bacteria was determined after overnight growth. The SBA was expressed as the reciprocal of the highest dilution of serum required to kill more than 50% of the bacteria relative to a no-serum control (49).

### Statistical analysis.

Statistical significance was calculated, using GraphPad Prism v.6.0, by unpaired Student *t* test or one-way ANOVA as indicated. A *P* value of <0.05 was considered statistically significant.

## Supplementary Material

Supplemental material
