# Supplementary material for: Neisseria cinerea Expresses a Functional Factor H Binding Protein Which Is Recognized by Immune Responses Elicited by Meningococcal Vaccines
Source: Infect Immun. 2017 Sep 20;85(10):e00305-17. doi: 10.1128/IAI.00305-17 (PMC5607398; doi:10.1128/IAI.00305-17)
Supplement: Supplemental material [file supp_85_10_e00305-17__index.html]

Supplemental material 

# Neisseria cinerea Expresses a Functional Factor H Binding Protein Which Is Recognized by Immune Responses Elicited by Meningococcal Vaccines

## Supplemental material

- Supplemental file 1 -

  Fig. S3. Sera from mice immunised with either V1.1 fHbp or Bexsero recognize fHbp from *Neisseria*.

  PDF, 277K
- Supplemental file 2 -

  Fig. S2. 5′-UTR of *fhbp* mRNA is conserved in *N. cinerea* CCUG 346T.

  PDF, 144K
- Supplemental file 3 -

  Fig. S1. Sequence alignment of *N. meningitidis* V1.1 fHbp with fHbp sequences of *N. cinerea* strains from http://pubmlst.org/.

  PDF, 359K
- Supplemental file 4 -

  Legends for Fig. 1 to 3.

  PDF, 299K
