# Supplementary material for: Neisseria cinerea Expresses a Functional Factor H Binding Protein Which Is Recognized by Immune Responses Elicited by Meningococcal Vaccines
Source: Infect Immun. 2017 Sep 20;85(10):e00305-17. doi: 10.1128/IAI.00305-17 (PMC5607398; doi:10.1128/IAI.00305-17)
Supplement: Supplemental material [file IAI.00305-17_zii999092159s1.pdf]

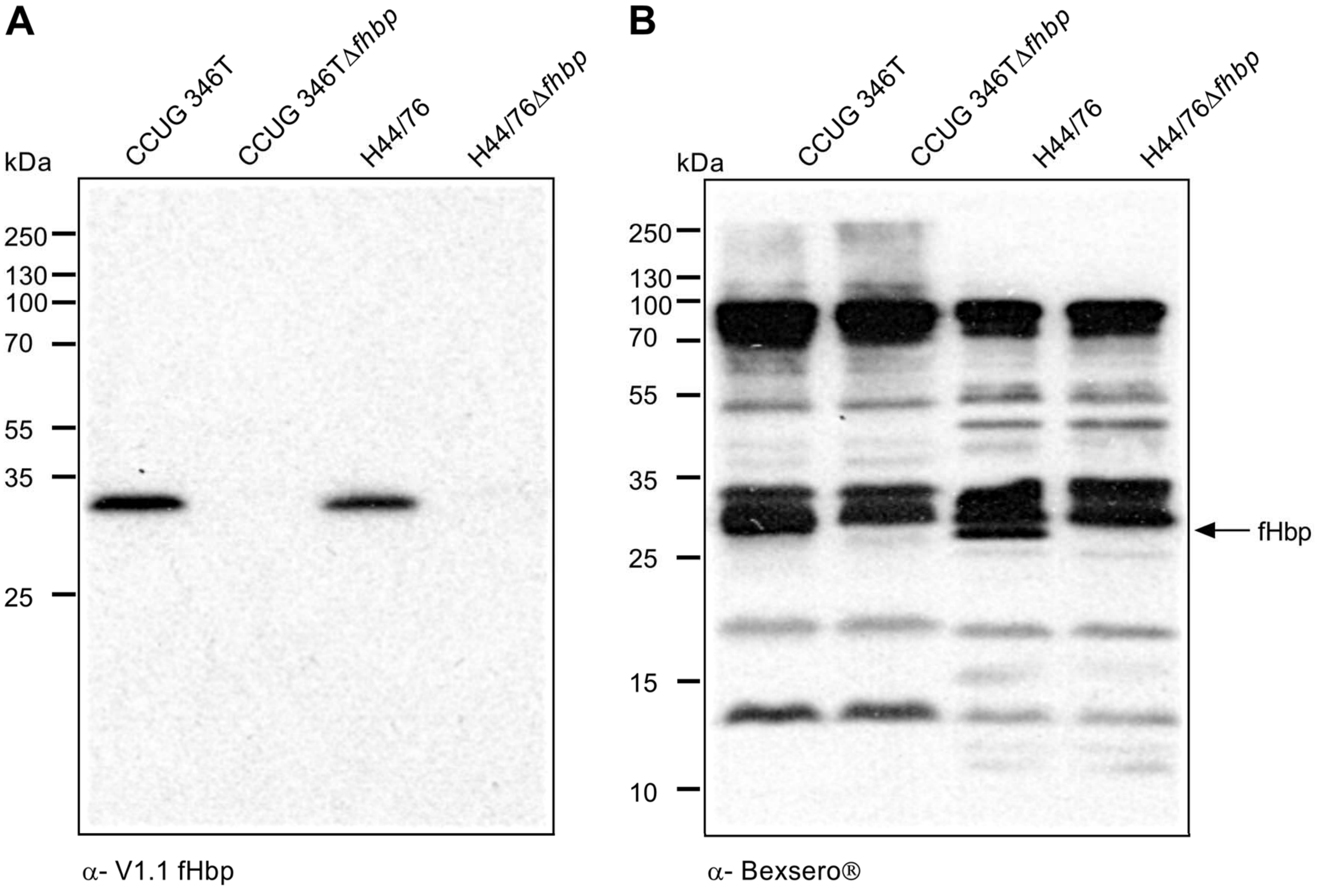
A
B
kDa
CCUG 346T
CCUG 346TΔ*fhbp*
H44/76
H44/76Δ*fhbp*
250
130
100
70
55
35
25
15
10
fHbp
α- V1.1 fHbp
α- Bexsero®
