# Supplementary material for: Neisseria cinerea Expresses a Functional Factor H Binding Protein Which Is Recognized by Immune Responses Elicited by Meningococcal Vaccines
Source: Infect Immun. 2017 Sep 20;85(10):e00305-17. doi: 10.1128/IAI.00305-17 (PMC5607398; doi:10.1128/IAI.00305-17)
Supplement: Supplemental material [file IAI.00305-17_zii999092159s2.pdf]

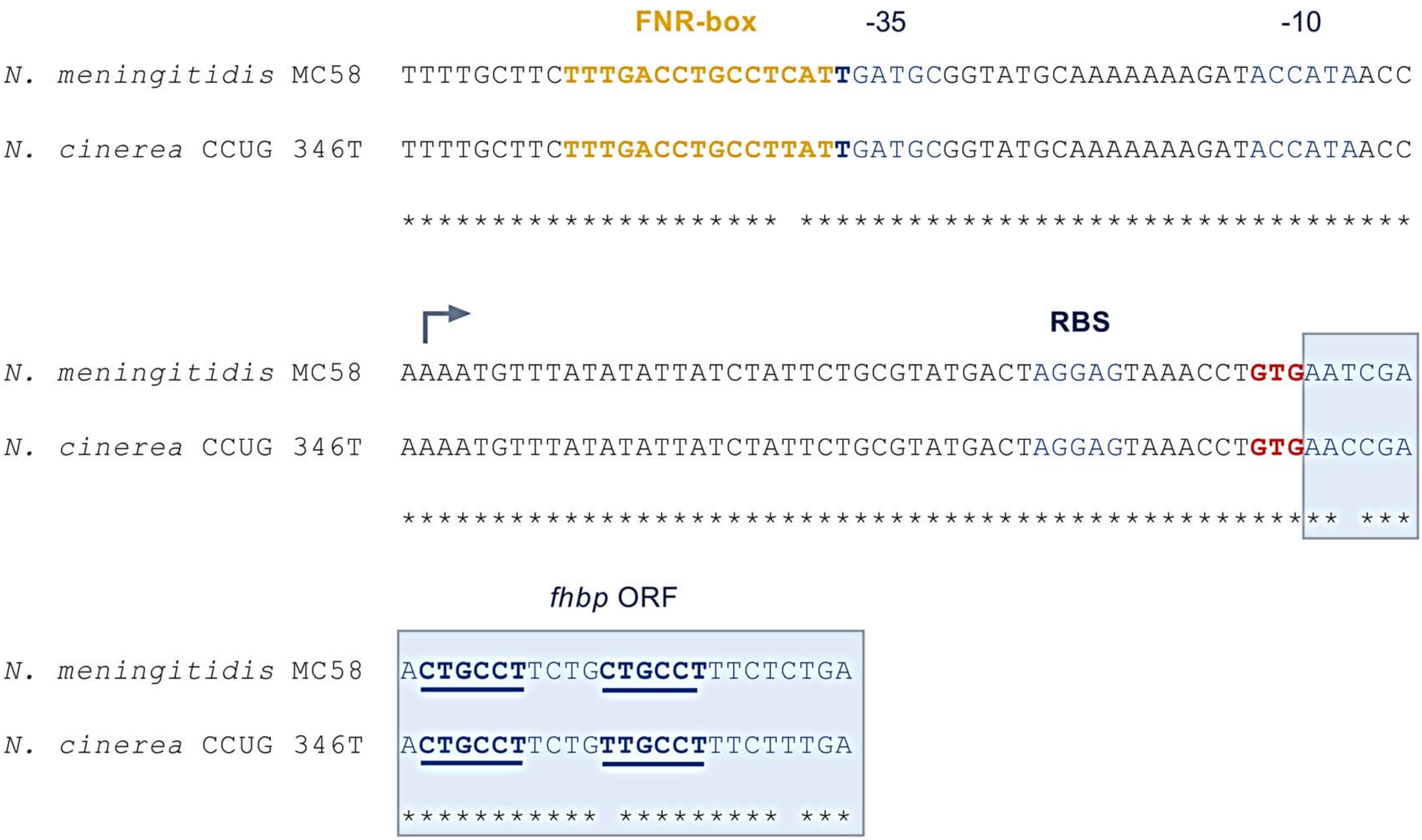
FNR-box
-35
-10
N. meningitidis MC58 TTTTGCTTCTTTGACCTGCCTCATTGATGCGGTATGCAAAAAAAGATACCATAACC
N. cinerea CCUG 346T TTTTGCTTCTTTGACCTGCCTTATTGATGCGGTATGCAAAAAAAGATACCATAACC
******************** *******************************
RBS
N. meningitidis MC58 AAAATGTTTATATATTATCTATTCTGCGTATGACTAGGAGTAAACCTGTGAATCGA
N. cinerea CCUG 346T AAAATGTTTATATATTATCTATTCTGCGTATGACTAGGAGTAAACCTGTGAACCGA
*** ***
fhbp ORF
N. meningitidis MC58 ACTGCCTTCTGCTGCCTTTCTCTGA
N. cinerea CCUG 346T ACTGCCTTCTGTTGCCTTTCTTTGA
*********** ********* ***
