# Supplementary material for: Neisseria cinerea Expresses a Functional Factor H Binding Protein Which Is Recognized by Immune Responses Elicited by Meningococcal Vaccines
Source: Infect Immun. 2017 Sep 20;85(10):e00305-17. doi: 10.1128/IAI.00305-17 (PMC5607398; doi:10.1128/IAI.00305-17)
Supplement: Supplemental material [file IAI.00305-17_zii999092159s3.pdf]

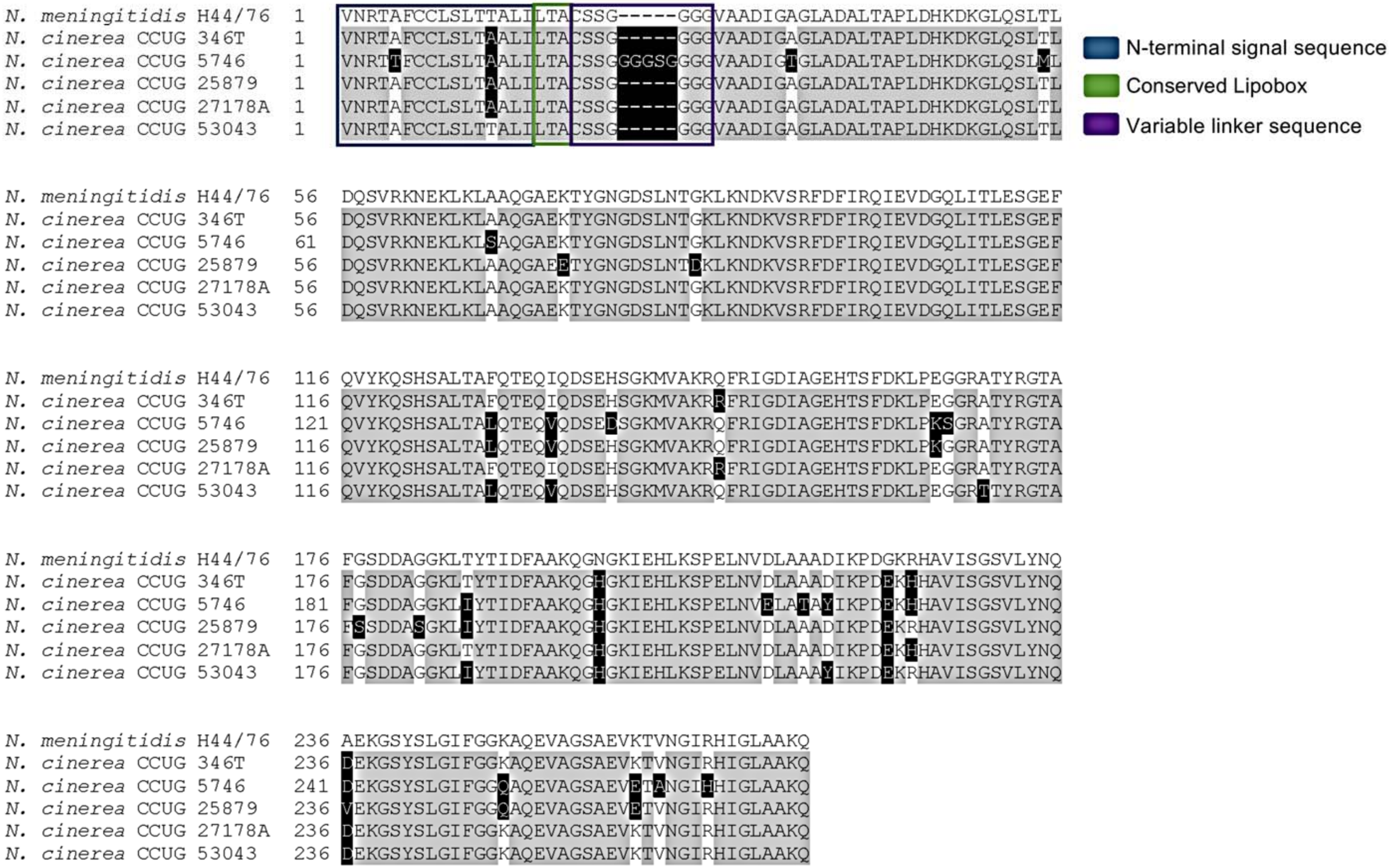
N. meningitidis H44/76 1 VNRTAFCCLSLTTALILTACSSG-----GGGVAADIGAGLADALTAPLDHKDKGLQSLTL
N. cinerea CCUG 346T 1 VNRTAFCCLSLTAALILTACSSG-----GGGVAADIGAGLADALTAPLDHKDKGLQSLTL
N. cinerea CCUG 5746 1 VNRTTFCCLSLTAALILTACSSGGGGSGGGGVAADIGTGLADALTAPLDHKDKGLQSLML
N. cinerea CCUG 25879 1 VNRTAFCCLSLTAALILTACSSG-----GGGVAADIGAGLADALTAPLDHKDKGLQSLTL
N. cinerea CCUG 27178A 1 VNRTAFCCLSLTAALILTACSSG-----GGGVAADIGAGLADALTAPLDHKDKGLQSLTL
N. cinerea CCUG 53043 1 VNRTAFCCLSLTTALILTACSSG-----GGGVAADIGAGLADALTAPLDHKDKGLQSLTL
N-terminal signal sequence
Conserved Lipobox
Variable linker sequence
N. meningitidis H44/76 56 DQSVRKNEKLKLAAQGAEKTYGNGDSLNTGKLKNDKVSRFDFIRQIEVDGQLITLESGEF
N. cinerea CCUG 346T 56 DQSVRKNEKLKLAAQGAEKTYGNGDSLNTGKLKNDKVSRFDFIRQIEVDGQLITLESGEF
N. cinerea CCUG 5746 61 DQSVRKNEKLKLSAQGAEKTYGNGDSLNTGKLKNDKVSRFDFIRQIEVDGQLITLESGEF
N. cinerea CCUG 25879 56 DQSVRKNEKLKLAAQGAEETYGNGDSLNTDKLKNDKVSRFDFIRQIEVDGQLITLESGEF
N. cinerea CCUG 27178A 56 DQSVRKNEKLKLAAQGAEKTYGNGDSLNTGKLKNDKVSRFDFIRQIEVDGQLITLESGEF
N. cinerea CCUG 53043 56 DQSVRKNEKLKLAAQGAEKTYGNGDSLNTGKLKNDKVSRFDFIRQIEVDGQLITLESGEF
N. meningitidis H44/76 116 QVYKQSHSALTAFQTEQIQDSEHSGKMVAKRQFRIGDIAGEHTSFDKLPEGGRATYRGTA
N. cinerea CCUG 346T 116 QVYKQSHSALTAFQTEQIQDSEHSGKMVAKRRFRIGDIAGEHTSFDKLPEGGRATYRGTA
N. cinerea CCUG 5746 121 QVYKQSHSALTALQTEQVQDSEDSGKMVAKRQFRIGDIAGEHTSFDKLPKSGRATYRGTA
N. cinerea CCUG 25879 116 QVYKQSHSALTALQTEQVQDSEHSGKMVAKRQFRIGDIAGEHTSFDKLPKGGRATYRGTA
N. cinerea CCUG 27178A 116 QVYKQSHSALTAFQTEQIQDSEHSGKMVAKRRFRIGDIAGEHTSFDKLPEGGRATYRGTA
N. cinerea CCUG 53043 116 QVYKQSHSALTALQTEQVQDSEHSGKMVAKRQFRIGDIAGEHTSFDKLPEGGRTTYRGTA
N. meningitidis H44/76 176 FGSDDAGGKLTYTIDFAAKQGNGKIEHLKSPELNVDLAAADIKPDGKRHAVISGSVLYNQ
N. cinerea CCUG 346T 176 FGSDDAGGKLTYTIDFAAKQGHGKIEHLKSPELNVDLAAADIKPDEKHHAVISGSVLYNQ
N. cinerea CCUG 5746 181 FGSDDAGGKLIYTIDFAAKQGHGKIEHLKSPELNVELATAYIKPDEKHHAVISGSVLYNQ
N. cinerea CCUG 25879 176 FSSDDASGKLIYTIDFAAKQGHGKIEHLKSPELNVDLAAADIKPDEKRHAVISGSVLYNQ
N. cinerea CCUG 27178A 176 FGSDDAGGKLTYTIDFAAKQGHGKIEHLKSPELNVDLAAADIKPDEKHHAVISGSVLYNQ
N. cinerea CCUG 53043 176 FGSDDAGGKLIYTIDFAAKQGHGKIEHLKSPELNVDLAAAYIKPDEKRHAVISGSVLYNQ
N. meningitidis H44/76 236 AEKGSYSLGIFGGKAQEVAGSAEVKTVNGIRHIGLAAKQ
N. cinerea CCUG 346T 236 DEKGSYSLGIFGGKAQEVAGSAEVKTVNGIRHIGLAAKQ
N. cinerea CCUG 5746 241 DEKGSYSLGIFGGQAQEVAGSAEVETANGIHHIGLAAKQ
N. cinerea CCUG 25879 236 VEKGSYSLGIFGGQAQEVAGSAEVETVNGIRHIGLAAKQ
N. cinerea CCUG 27178A 236 DEKGSYSLGIFGGKAQEVAGSAEVKTVNGIRHIGLAAKQ
N. cinerea CCUG 53043 236 DEKGSYSLGIFGGKAQEVAGSAEVKTVNGIRHIGLAAKQ
