# Supplementary material for: Neisseria cinerea Expresses a Functional Factor H Binding Protein Which Is Recognized by Immune Responses Elicited by Meningococcal Vaccines
Source: Infect Immun. 2017 Sep 20;85(10):e00305-17. doi: 10.1128/IAI.00305-17 (PMC5607398; doi:10.1128/IAI.00305-17)
Supplement: Supplemental material [file IAI.00305-17_zii999092159s4.pdf]

Supplementary Figure 1. Sequence alignment of *N. meningitidis* V1.1 fHbp with fHbp sequences of *N. cinerea* strains from (http://pubmlst.org/). Black boxed residues show sequence variation of fHbp and white boxed residues show identical residues compared with *N. meningitidis* V1.1 fHbp sequence. Grey boxed residues show residues conserved in all strains. Sequence analysis indicates a conserved signal motif (blue box), lipobox (green box) and variable linker sequence (purple box)

Supplementary Figure 2. 5'-UTR of *fhbp* mRNA is conserved in *N. cinerea* CCUG 346T. Sequence alignment of *fhbp* 5'-UTR of *N. meningitidis* MC58 and *N. cinerea* CCUG 346T, *fhbp* promoter (blue arrows), the FNR box (yellow), the -35 and -10 sequences with the RBS (shown blue), and the start codon, red, GTG) are indicated. Blue boxed residues indicate the ORF of fHbp. Bold, blue underlined residues in the ORF indicate putative α-RBS 1 and 2.

Supplementary Figure 3. Sera from mice immunised with either V1.1 fHbp or Bexsero® recognise fHbp from *Neisseria.* Proteins recognised in whole cell extracts of *N. meningitidis*, H44/76 and H44/76Δ*fhbp,* as well as *N. cinerea*, CCUG 346T and CCUG 346TΔ*fhbp* by sera from mice immunised with either V1.1 fHbp **(A)** or Bexsero® **(B)** as determined by Western blot analysis. Mice immunised with Bexsero® recognise multiple proteins in *Neisseria* whole cell extracts, due to the other components contained in the vaccine, the molecular weight of fHbp (27kDa) is indicated.
